# Supplementary material for: Endoplasmic Reticulum-Targeting Two-Photon Fluorescent Probe for CYP1A Activity and Its Imaging Application in Endoplasmic Reticulum Stress
Source: Molecules. 2023 Apr 14;28(8):3472. doi: 10.3390/molecules28083472 (PMC10143423; doi:10.3390/molecules28083472)
Supplement: Supplementary file 1 [file molecules-28-03472-s001.zip › molecules-2237976-supplementary.pdf]

Supplementary Material for

Endoplasmic Reticulum-Targeting Two-Photon Fluorescent Probe for CYP1A Activity and Its Imaging Application in Endoplasmic Reticulum Stress

Chao Shi[1,2,†], Yan Wang[2,†], Xiangge Tian[1], Xia Lv[2], Yue An[1], Jing Ning[1,2,*], Xiulan Xin[3], Li Dai[1,*], Xiaochi Ma[1] and Lei Feng[1,4,5*]

[1]Second Affiliated Hospital, Dalian Medical University, Dalian 116023, China
[2]College of Pharmacy, Dalian Medical University, Dalian 116044, China
[3]College of Bioengineering, Beijing Polytechnic, Beijing 100029, China
[4]School of Chemistry and Chemical Engineering, Henan Normal University, Xinxiang 453007, China
[5]Key Laboratory of Emergency and Trauma of Ministry of Education, Hainan Medical University, Haikou 571199, China

[*]Correspondence: ningjing0626@163.com (J.N.); daily21st@aliyun.com (L.D.); leifeng@dmu.edu.cn (L.F.)

[†]These authors made equal contributions to this work.

Table of Contents

Supplementary Figure S1. 1

Supplementary Figure S2. 1

Supplementary Figure S3. 2

Supplementary Figure S4. 2

Supplementary Figure S5. 3

Supplementary Table S1. 3

NMR and MS Spectra. 4

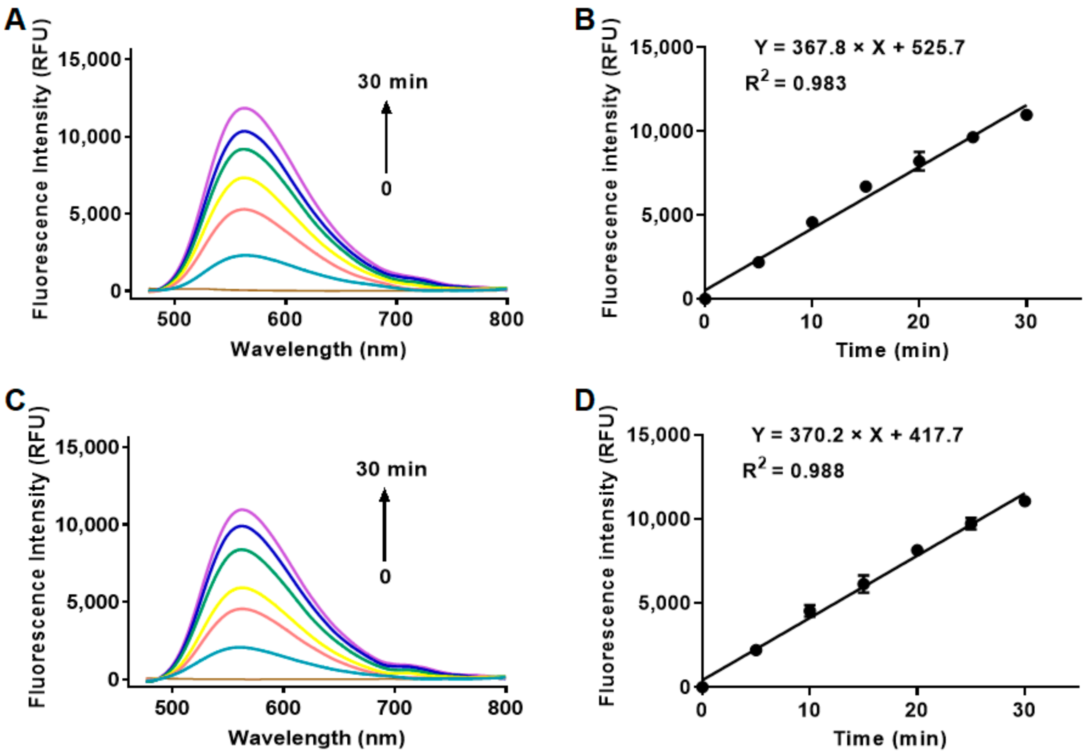

Supplementary Figure S1. Fluorescence spectra of **ERNM** that incubated with 7.5 nM CYP1A1 (A) and 7.5 nM CYP1A2 (C) with the prolonging of time, respectively. Fluorescence intensity of **ERNM** at 558 nm that incubated with CYP1A1 (B) and CYP1A2 (D) for different duration. λ_{ex} = 450 nm. Data in Figure B and D are shown as the mean ± S.D.

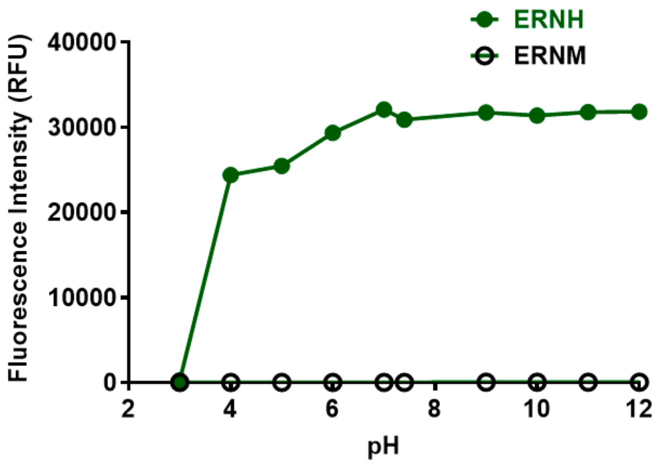

Supplementary Figure S2. The effects of pH values on the fluorescence intensity of **ERNM** and its metabolite **ERNH** (2.5 μM).

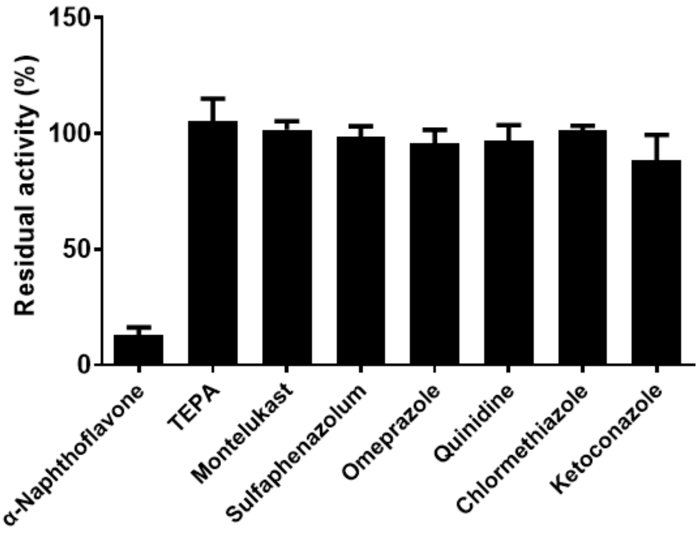

Supplementary Figure S3. Inhibitory effects of selective CYP inhibitors on **ERNM** *O*-demethylation in human liver microsomes. TEPA, N,N',N''-triethylene thiophosphoramide.

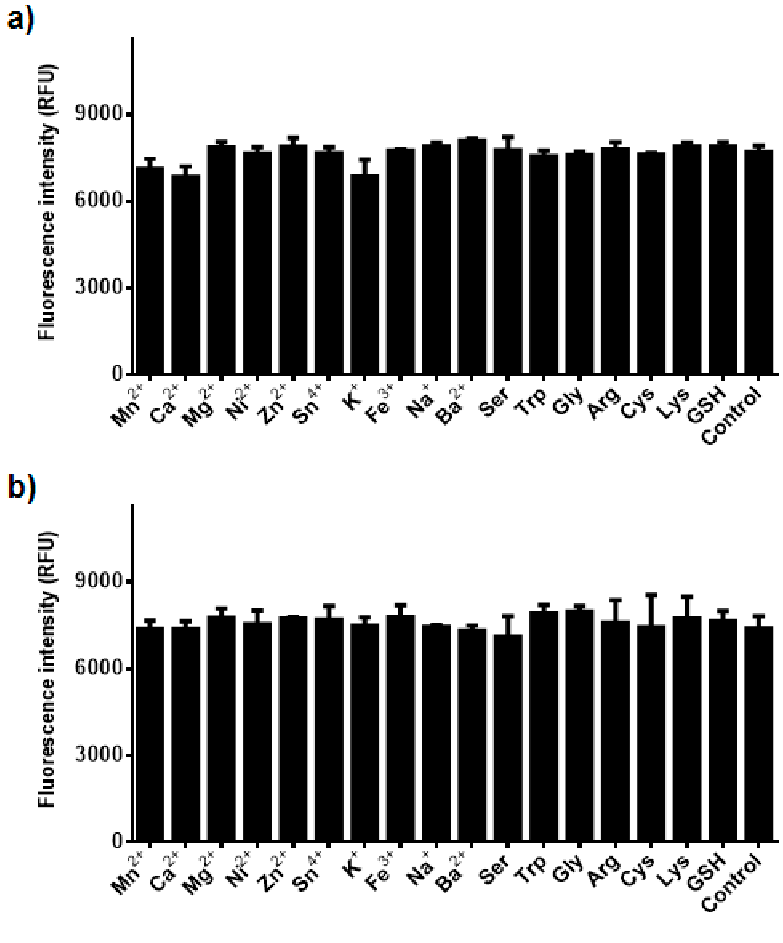

Supplementary Figure S4. Fluorescence responses of **ERNM** towards CYP1A1 (a) and CYP1A2 (b) in the presence of various analytes.

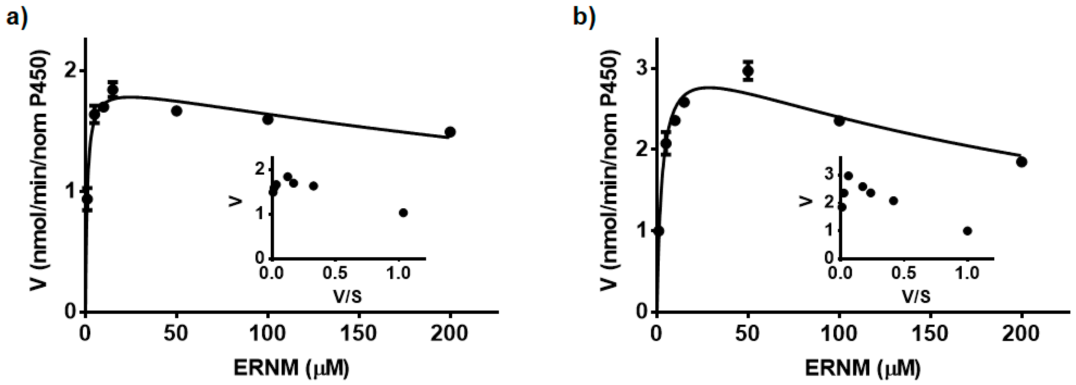

Supplementary Figure S5. Kinetic plots of **ERNM** *O*-demethylation in CYP1A1 (a) and CYP1A2 (b). The corresponding Eadie-Hofstee plot is shown as an inset.

Table S1. Kinetic parameters of **ERNM** *O*-demethylation determined in CYP1A1 and CYP1A2.

Enzyme sources	V_{max} (nmol/min/nmol p450)	K_m (μM)	K_{si} (μM)
CYP1A1	1.92 ± 0.04	1.0 ± 0.1	611.3 ± 93.8
CYP1A2	3.32 ± 0.15	2.9 ± 0.5	281.2 ± 50.2

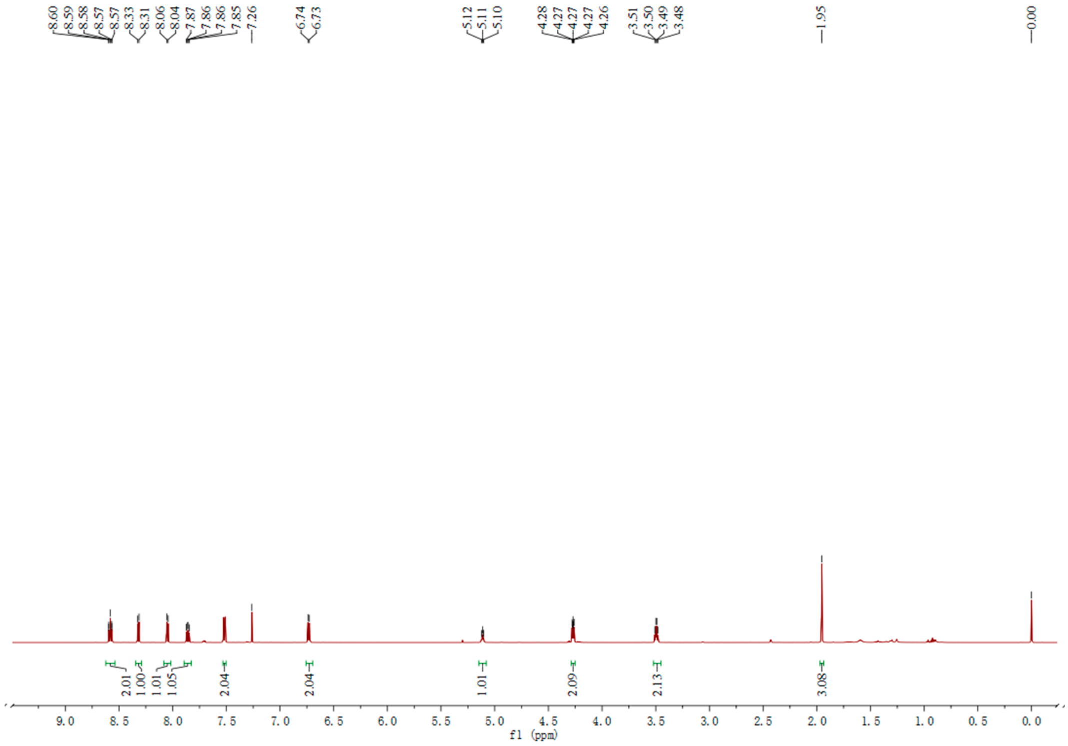

Supplementary Figure S6. ^{1}H NMR of **ERNBr**

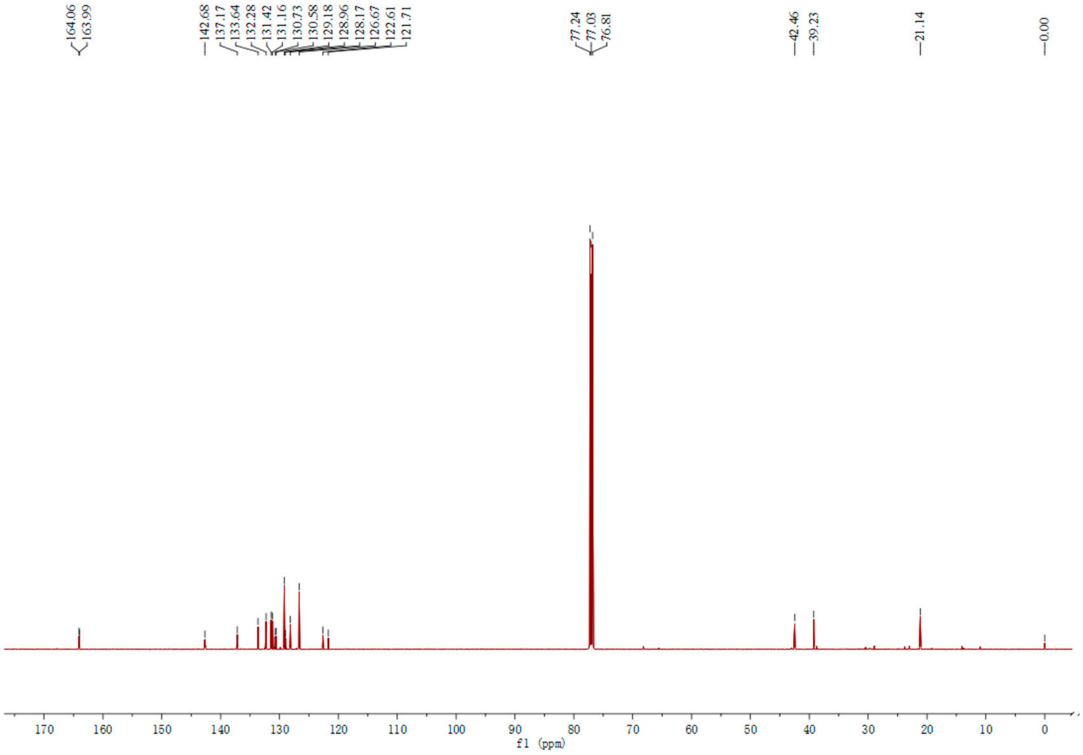

Supplementary Figure S7. ^{13}C NMR of **ERNBr**

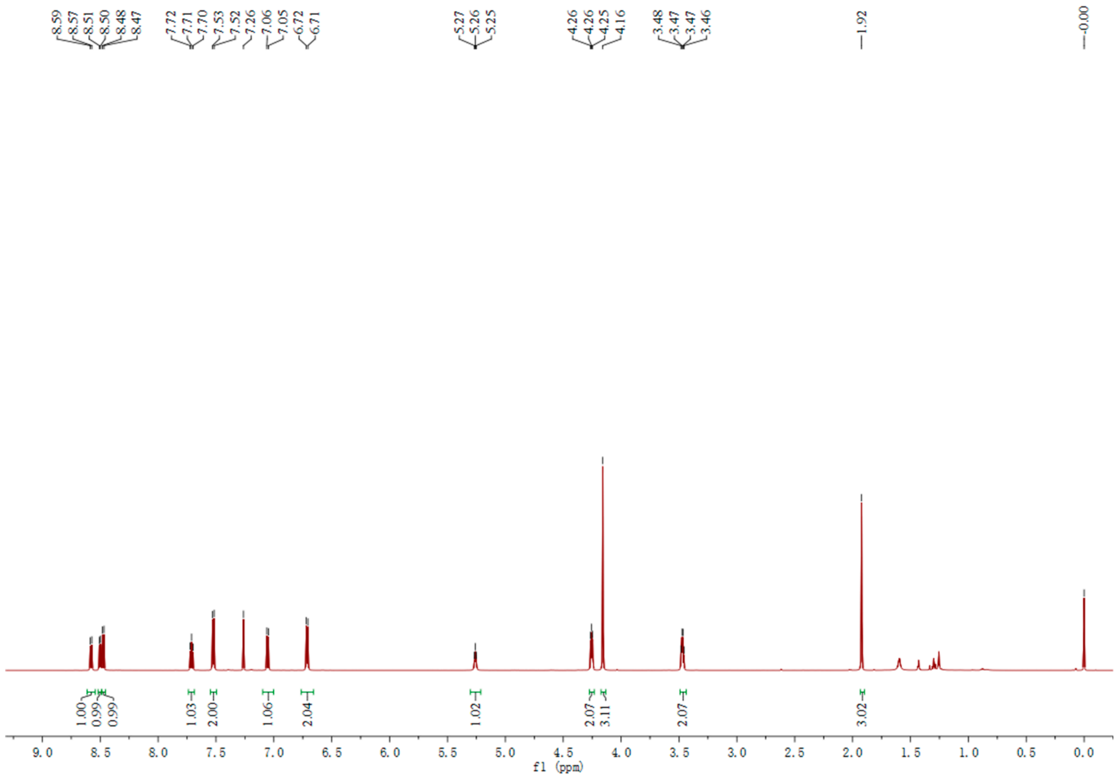

Supplementary Figure S8. ^{1}H NMR of **ERNM**

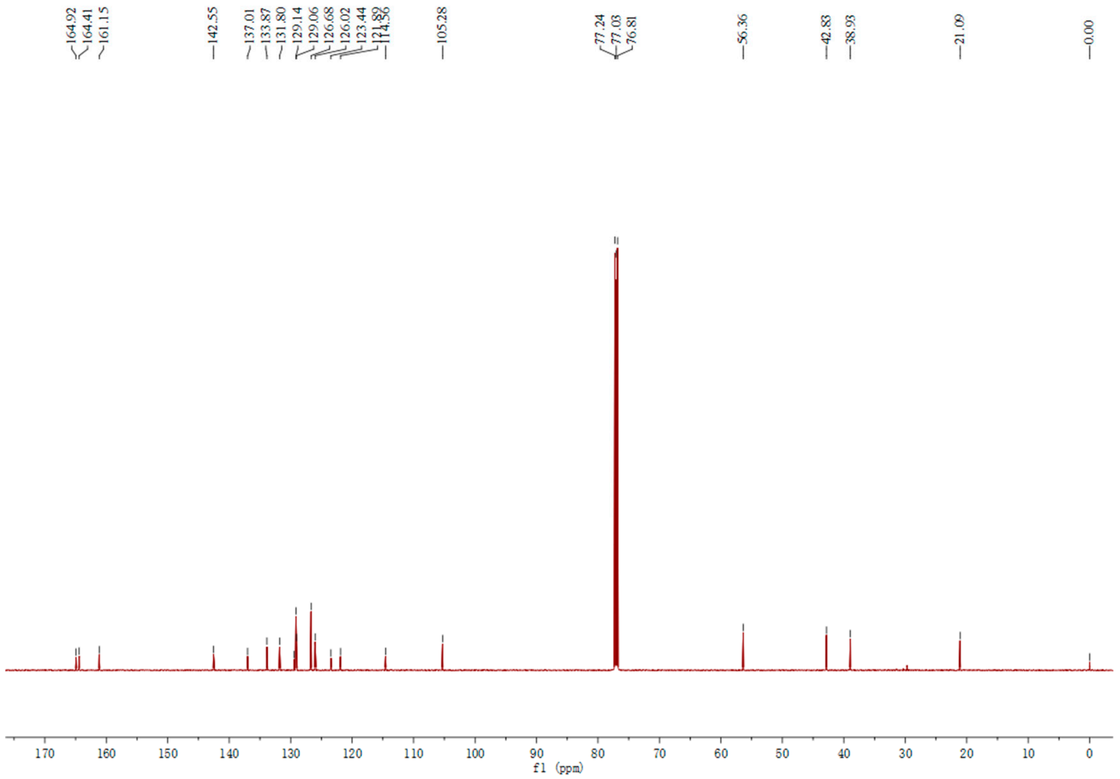

Supplementary Figure S9. ^{13}C NMR of **ERNM**

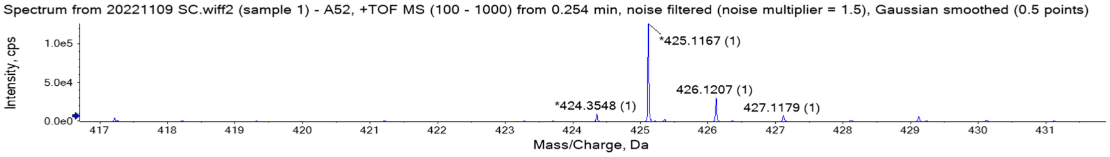

Supplementary Figure S10. HRMS spectrum of **ERNM**
